# VirBot: an RNA viral contig detector for metagenomic data

**DOI:** 10.1093/bioinformatics/btad093

**Published:** 2023-02-16

**Authors:** Guowei Chen, Xubo Tang, Mang Shi, Yanni Sun

**Affiliations:** Department of Electrical Engineering, City University of Hong Kong, Kowloon, Hong Kong SAR, China; Department of Electrical Engineering, City University of Hong Kong, Kowloon, Hong Kong SAR, China; School of Medicine, Shenzhen Campus of Sun Yat-sen University, Shenzhen, China; Department of Electrical Engineering, City University of Hong Kong, Kowloon, Hong Kong SAR, China

## Abstract

**Summary:**

Without relying on cultivation, metagenomic sequencing greatly accelerated the novel RNA virus detection. However, it is not trivial to accurately identify RNA viral contigs from a mixture of species. The low content of RNA viruses in metagenomic data requires a highly specific detector, while new RNA viruses can exhibit high genetic diversity, posing a challenge for alignment-based tools. In this work, we developed VirBot, a simple yet effective RNA virus identification tool based on the protein families and the corresponding adaptive score cutoffs. We benchmarked it with seven popular tools for virus identification on both simulated and real sequencing data. VirBot shows its high specificity in metagenomic datasets and superior sensitivity in detecting novel RNA viruses.

**Availability and implementation:**

https://github.com/GreyGuoweiChen/RNA_virus_detector

**Supplementary information:**

Supplementary data are available at *Bioinformatics* online.

## 1 Introduction

RNA viruses have high diversity and can infect a large variety of eukaryotes from planktons to humans. With an estimated number of over 100 million eukaryotic viruses, <0.1% RNA viruses are recorded (Zhang *et al.*, 2019). Both metagenomic and viral metagenomic sequencing data are primary sources for virus discovery (Greninger, 2018). However, both types of data can be loaded with organisms of different origins. To identify RNA viruses from the heterogeneous data, generic alignment tools and targeted virus detection tools have been adopted in different applications (Buchfink *et al.*, 2015; Guo *et al.*, 2021; Menzel *et al.*, 2016; Ren *et al.*, 2017; Steinegger and Söding, 2017; Wood *et al.*, 2019). But, their performance deteriorates on detecting short RNA viral contigs from new habitats.

In this work, we introduce VirBot, an easy-to-use yet effective RNA virus detection tool for metagenomic data. It takes assembled contigs as input and detects ones from RNA viruses. In addition, it outputs the taxonomic labels for the detected RNA viruses. We validated VirBot in various scenarios and benchmark it with other RNA virus detection tools. In samples that only contain novel RNA viruses, VirBot achieves higher recall than other tools. And VirBot also demonstrates high specificity in metagenomic data that only contains a small number of RNA viruses.

## 2 Materials and methods

**Fig. 1.**
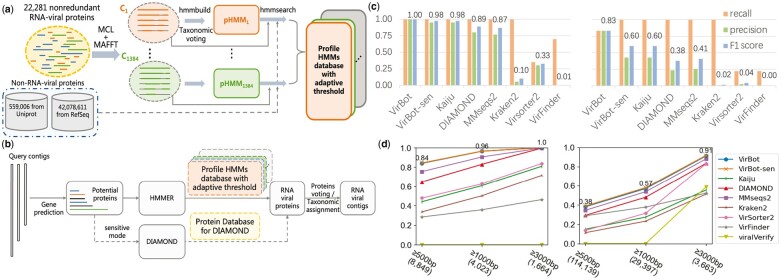
(**a**) Construction of the RNA viral pHMMs database. (**b**) Sketch of the key components of VirBot. (**c**) Detection performance on simulated data: ERR1992810 (left) and ERR2185279 (right). (**d**) Recall on RNA viral datasets: RNA phages dataset (left) and marine water RNA virome dataset (right)

VirBot detects RNA viruses using our self-constructed protein domain families and their customized score cutoffs. As shown by a recent analysis (Yuan *et al.*, 2022), RNA viruses have a better conservation in their protein sequences than their nucleic acid sequences. Some proteins, including RdRps, capsid proteins and envelope proteins, have been widely applied to study the evolution and phylogeny of RNA viruses (Shi *et al.*, 2018). Our previous work leveraging RdRp (Tang *et al.*, 2022) has contributed to sensitive viral read identification. However, using RdRp itself is limited because short contigs may not overlap with RdRp genes. Thus, we construct our own RNA viral protein families by clustering 22 281 non-redundant RNA viral proteins derived from available RNA virus and determine the family-specific cut-off using 814 991 non-RNA viral proteins from Uniprot and RefSeq (Fig. 1a). Finally, there are 1384 protein families and 3927 singletons, which only contain one protein sequence. We encode all the families using pHMMs to enhance remote homology search and provide a preliminary taxonomic prediction based on the known label of each sequence in the protein family. Unlike previous tools, we did not use public protein domain database such as Pfam because it is not optimized for RNA viruses. For example, RdRp is an essential protein responsible for RNA replications. When searching with the keyword ‘rdrp’ in Pfam (35.0), it returns 73 pHMMs, and only 13 are constructed from viral RdRp. The limited number of RdRp families specifically constructed for RNA viruses does not comply with the diversity and abundance of RNA viruses. The details about the protein family database construction, the determination of the adaptive cutoffs, and the taxonomic label assignment for each family can be found in Supplementary Figure S1 and Supplementary Section S2. VirBot’s query stage is sketched in Figure 1b, where an input contig will be classified based on the result of majority vote on its predicted proteins against our protein family database and the singletons.

## 3 Results

We validated VirBot in five scenarios and compared its performance against seven popular tools. The commands and parameters of the tools are summarized in Supplementary Tables S1 and S2. More details about the experiments and the taxonomic assignment evaluation can be found in Section Result of the Supplementary File.

***Simulated metagenomic data.*** We first evaluated VirBot on two semi-synthetic marine metagenomic samples (NCBI SRA ID: ERR1992810 and ERR2185279) constructed from 82 eukaryotes, 365 prokaryotes and DNA/RNA viruses (Mitchell *et al.*, 2019). As shown in Figure 1c, VirBot showed the highest precision and F1 scores in these samples. More details about the data and the other results on contigs of different lengths are shown in Supplementary Table S3 and Supplementary Figures S2 and S3.

***Identifying new RNA virus.*** To assess the ability of VirBot in identifying new RNA viruses, we used three datasets that contain newly sequenced RNA viruses. One comprises 8849 RNA phages that were barely detected before, while eukaryotic viruses dominate known RNA viruses. The second dataset is an RNA virome sample sequenced from marine water containing 114 139 RNA viral sequences. The third is made up of 5504 non-redundant sequences containing viral RdRp region detected from the global ocean resources. As shown in Figure 1d, Supplementary Figure S4, VirBot has the best recall among all tools on contigs of different length ranges.

***Real metagenomic data.*** We tested VirBot on 21 human clinical samples for pathogen detection. VirBot successfully retrieved all the reported RNA viral pathogens and achieved an average recall of 99.3% and precision of 99.2% among different groups. The result is shown in Supplementary Table S5.

## 4 Conclusion

In this work, we constructed a comprehensive protein family database for RNA viruses and derived the adaptive bit score cutoff using a large number of non-RNA viral proteins. We developed VirBot based on these RNA viral pHMMs for fast and more sensitive RNA virus detection in metagenomic data. Our pHMMs are designed specifically for RNA virus and show higher sensitivity than other HMM-based tools (Fig. 1d, Supplementary Fig. S4 and Supplementary Table S4). Compared with using a fixed cut-off (recommended E-value), VirBot improved the specificity from 8.8% to 97.4% in the simulated data, which is shown in Supplementary Table S6.

## Supplementary Material

btad093_Supplementary_Data

## Data Availability

The source code of VirBot and the data are available at https://github.com/GreyGuoweiChen/RNA_virus_detector.

## References

[btad093-B1] Buchfink B. et al (2015) Fast and sensitive protein alignment using DIAMOND. Nat. Methods, 12, 59–60. 25402007 10.1038/nmeth.3176

[btad093-B2] Greninger A.L. (2018) A decade of RNA virus metagenomics is (not) enough. Virus Res., 244, 218–229. 29055712 10.1016/j.virusres.2017.10.014 PMC7114529

[btad093-B3] Guo J. et al (2021) VirSorter2: a multi-classifier, expert-guided approach to detect diverse DNA and RNA viruses. Microbiome, 9, 1–13. 33522966 10.1186/s40168-020-00990-y PMC7852108

[btad093-B4] Menzel P. et al (2016) Fast and sensitive taxonomic classification for metagenomics with kaiju. Nat. Commun., 7, 1–9. 10.1038/ncomms11257 PMC4833860 27071849

[btad093-B5] Mitchell A. et al (2019) ELIXIR-EXCELERATE D6.3: Report Describing a Set of Tools, Pipelines and Search Engine for Interrogation of Marine Metagenomic Data. Zenodo. 10.5281/zenodo.3228302.

[btad093-B6] Ren J. et al (2017) VirFinder: a novel k-mer based tool for identifying viral sequences from assembled metagenomic data. Microbiome, 5, 1–20. 28683828 10.1186/s40168-017-0283-5 PMC5501583

[btad093-B7] Shi M. et al (2018) The evolutionary history of vertebrate RNA viruses. Nature, 556, 197–202. 29618816 10.1038/s41586-018-0012-7

[btad093-B8] Steinegger M. , Söding J. (2017) MMseqs2 enables sensitive protein sequence searching for the analysis of massive data sets. Nat. Biotechnol., 35, 1026–1028. 29035372 10.1038/nbt.3988

[btad093-B9] Tang X. et al (2022) RdRp-based sensitive taxonomic classification of RNA viruses for metagenomic data. Brief. Bioinform., 23, bbac011. 35136930 10.1093/bib/bbac011 PMC8921650

[btad093-B10] Wood D.E. et al (2019) Improved metagenomic analysis with Kraken 2. Genome Biol., 20, 1–13. 31779668 10.1186/s13059-019-1891-0 PMC6883579

[btad093-B11] Yuan W.-G. et al (2022) A discussion of RNA virus taxonomy based on the 2020 ICTV report. Front. Microbiol., 13, 960465. 36312925 10.3389/fmicb.2022.960465 PMC9615923

[btad093-B12] Zhang Y.-Z. et al (2019) Expanding the RNA virosphere by unbiased metagenomics. Annu. Rev. Virol., 6, 119–139. 31100994 10.1146/annurev-virology-092818-015851

