# Supplementary material for: VirBot: an RNA viral contig detector for metagenomic data
Source: Bioinformatics. 2023 Feb 16;39(3):btad093. doi: 10.1093/bioinformatics/btad093 (PMC9978584; doi:10.1093/bioinformatics/btad093)
Supplement: btad093_Supplementary_Data [file btad093_supplementary_data.pdf]

VirBot: an RNA viral contig detector for metagenomic data

Guowei Chen, Xubo Tang, Mang Shi, and Yanni Sun

Supplementary materials

1 Summary

This supplementary file provides detailed methods and experimental results behind our RNA virus detection tool, VirBot.

2 Material and Method

2.1 Framework of VirBot

The complete pipeline of VirBot is illustrated in Fig. S1(a). Compared to Fig. 1 in the main draft, we added the schematic diagrams about determining the adaptive cutoff and taxonomic label for each protein family.

During the query stage, Prodigal [1] is applied to identify genes and translate them into proteins for each contig. And VirBot decides whether the contig originates from RNA virus based on each protein's prediction. Because of the small viral genome size and their high density of coding regions, even short viral contigs assembled from metagenomic data often contain genes, which makes this simple method applicable to more complex samples. Moreover, based on the matches against our pHMMs, VirBot assigns taxa to the detected contigs.

2.2 Protein sequences from RNA viruses and other species

We downloaded 28,998 RNA-viral proteins from NCBI, which were deduplicated to 22,281 non-redundant proteins by CD-HIT [2]. We then input the non-redundant RNA viral proteins to the clustering step. To determine the adaptive cutoff, we downloaded 10,223 DNA-viral and 548,783 cellular proteins from Swiss_Prot. Considering that bacterial sequences are often detected in the same sample as RNA viruses, we additionally downloaded 58,838,813 bacterial proteins from NCBI RefSeq by genome_updater [3] as the negative samples. We deduplicated the bacterial proteins by MMseqs2 in linclust mode and got 42,078,611 nonredundant bacterial proteins.

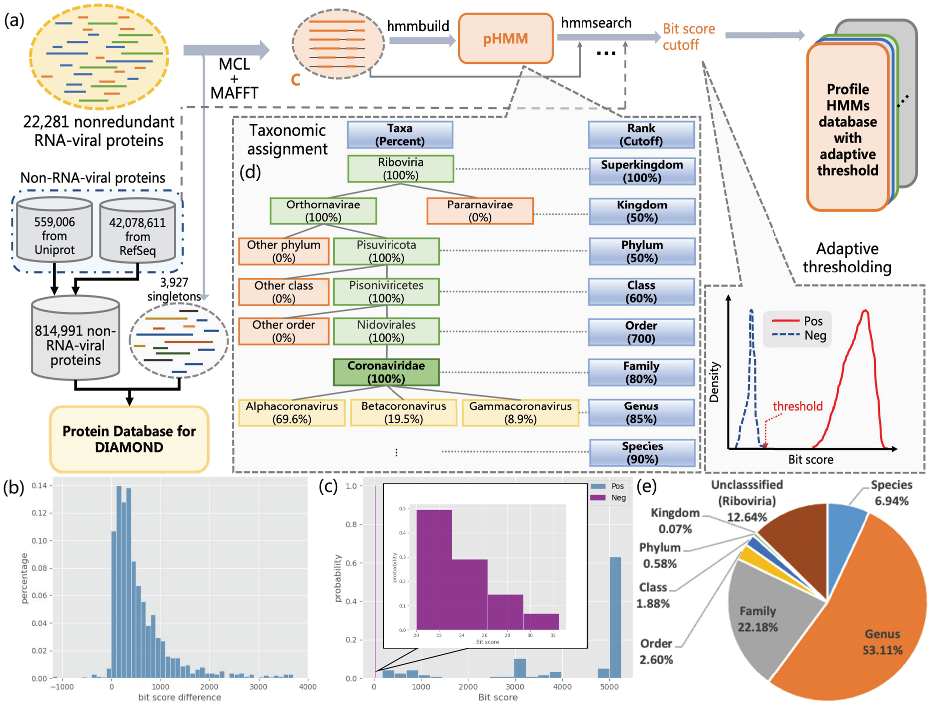

Figure S1: (a) Construction of the reference database. C: a protein family. (b) The histogram of the bit score differences for our pHMMs. X-axis: The difference between the smallest bit score of RNA viral proteins and the largest bit score of non-RNA viral proteins for each pHMM; Y-axis: The percentage of pHMMs. (c) Bit score distribution of a representative pHMM that is constructed from 117 RNA-viral proteins. Due to space limitation, we only show the distribution for scores above 20. There are 192 out of 42,637,617 non-RNA-viral proteins matched to this pHMM (purple bars). (d) A representative example of taxonomic assignment using a pHMM. (e) The distribution of taxonomic rank of the pHMM database.

Program	Non-default parameters
cd-hit	-c 0.99 -s 0.1
	The deduplicated sequences should share at least 99% identity with the representative sequences and its length should be no shorter than 10%.
diamond	–query-cover 0.1 –subject-cover 0.1
	The protein alignment is done between sequences whose lengths differ by no more than ten times.
mcxload	–stream-neg-log10 -stream-tf 'ceil(300)'
	Set the edge weights by transforming the E-value of alignment to negative log10 scale. The maximum weight is 300.
mcl	-I 2.0
	Set the inflation of markov clustering as 2.
mafft	–maxiterate 1000 –genafpair
	Use genafpair mode for MAFFT and set the maximum iteration as 1000 to improve the accuracy of MSA.

Table S1: Parameters for building the pHMMs.

2.3 RNA-viral pHMM Construction

We built protein families and their pHMMs using RNA viral proteins and then decided the best bit score cutoff using the negative samples (i.e. proteins from other species). Previously, some pHMM databases have been compiled for functional annotation [4] and taxonomic classification [5]. Pfam is one of the most well-known database. Both VirSorter2 and viralVerify integrated Pfam for virus identification. However, Pfam is not optimized for protein domains in RNA viruses. For example, RdRp is an essential protein responsible for RNA replications. When searching the keyword "rdrp" in Pfam (35.0), it returns 73 pHMMs, and only 13 are constructed from viral RdRp. The limited number of RdRp families specifically constructed for RNA viruses does not comply with the diversity and abundance of RNA viruses.

To build the pHMMs, we first conducted pairwise alignment for 22,281 non-redundant RNA viral proteins with DIAMOND. Then we used a graph-based clustering method, Markov clustering (MCL) [6], to cluster proteins into protein families based on the alignments' E-values. This step returned 4,572 clusters, and we only kept 1,384 clusters that contained more than two proteins. Finally, we generated the multiple sequence alignment (MSA) for each cluster using MAFFT [7] and constructed the corresponding pHMM using HMMER3 [8]. We denoted sequences from cluster i by C_i, and pHMM$_i$ by P_i. The programs' parameters are shown in Table S1.

To enrich the RNA viral protein families, VirBot and VirSorter2 built their own pHMMs and achieved 239 and 228 RdRp families, respectively. Unlike VirSorter2, VirBot includes other proteins such as retrotranscriptase (RT), capsid proteins, and envelope proteins, which play an essential role in the survival and reproduction of RNA viruses. The inclusion of more proteins is expected to improve the sensitivity of VirBot.

2.4 Taxonomic assignment

VirBot provides preliminary taxonomic prediction for the detected contigs by analyzing the composition of each protein family. In a protein family, we counted the taxonomic label of each RNA viral protein and recorded the most popular ones across different levels/ranks. From species to realm (Riboviria), if the percentage of sequences with the dominant label surpasses the customized cutoff, we assign this dominant label to the corresponding pHMM. If a pHMM has assigned labels at multiple ranks with consistency, only the label at the lowest rank is kept. An example is shown in Fig. S1(d). The example pHMM has member proteins with the same label till the family level. However, its label composition at the genus level cannot reach the specified cutoff, and thus it is assigned as Coronaviridae but not a genus. To prevent the prediction from being overconfident, we set different cutoffs for different levels. Overall, the lower ranks have more stringent cutoffs. The distribution of pHMMs' rank is shown in Fig. S1(e), where 1,138 pHMMs (82.2%) can be assigned to family or lower ranks and 175 (12.6%) are "unassigned". The reason behind these unassigned pHMMs is that their component proteins are mostly from unclassified RNA viruses. For instance, the protein family C_5 consists of 121 RNA viral proteins, and 81 are unclassified "hypothetical proteins".

Once we decide the taxonomic label of each pHMM, we provide two modes for the contigs' taxonomic assignment, TOP mode and LCA mode. In TOP mode, VirBot assigns a taxonomic label to a contig according to its best matched pHMM. For example, if best matched pHMM has a label 'Coronaviridae', the contig will be assigned as 'Coronaviridae'. This simple strategy is the default mode in VirBot. In LCA mode, VirBot considers the taxa of all matched pHMMs of a contig and will assign it to the lowest common ancestor of the pHMMs.

2.5 Adaptive threshold

Although a default E-value is often used as the criterion for pHMM matching, we found that using a unified E-value or bit score cutoff can lead to false positive hits for some protein families. We analyzed the bit score distribution of both positive (RNA-viral) and negative (non-RNA-viral) proteins, based on which we selected the adaptive cutoffs τ_i for each family model P_i. A pHMM with reasonable distinguishing power should have minimum overlap between the score distributions from positive and negative samples. Let the largest negative score be MAX_i^{neg} and the smallest positive score be MIN_i^{pos} for the ith family, respectively. We plot the distribution of $MIN_i^{pos} - MAX_i^{neg}$ for all C_i in Fig. S1(c), which shows that most pHMMs (1366 out of 1384) have a clear gap between MAX_i^{neg} and MIN_i^{pos} with the gap size peaking between 100 and 400. A representative example of the pHMM's score distribution is plotted in Fig. S1(d), where the negative samples are accumulated around the low score region (<30). Distribution overlap happens to only 19 pHMMs, resulting from the shared domains between RNA virus and other species. Thus, a majority of

our protein families can distinguish positive inputs from negative ones.

Because a majority of our pHMMs produce bit scores below 30 for negative proteins, we empirically set the significance bit score *threshold* to 30, which is larger than the recommended cutoff by HMMER. Any sequences with bit score larger than the *threshold* would be regarded to share homologous domains with the pHMMs instead of hitting by chance. If MIN_i^{pos} is smaller than 30, we update the threshold as MIN_i^{pos}. We got 319 families with MAX_i^{neg} above 30, representing blurred boundaries between RNA viruses and negative samples in these families. To improve the specificity, we set the minimum threshold as MAX_i^{neg}, which records the highest score for the negative data. Thus, we set τ_i as $max(MAX_i^{neg}, threshold)$ for these families. In summary, the above relation can be encoded by Equation 1.

$$\tau_i = max(MAX_i^{neg}, min(MIN_i^{pos}, 30)) \tag{1}$$

To predict whether a query contig is RNA virus or not, VirBot searches every potential protein of the contig. We apply a gene-finding tool such as Prodigal to translate the genes in the contig into proteins, which are used as queries to our pHMM database. Considering that new proteins might present in a newly sequenced RNA virus, we do not require each protein to match our pHMMs. Instead, we empirically determine a cutoff using the percentage of matched proteins. According to NCBI RefSeq, a typical RNA virus has small genome (2∼33 kbps) and encodes at most 16 proteins, so we require at least one match of RNA-viral protein every 16 proteins.

2.6 Reference database for the DIAMOND module

Besides the proteins in the pHHMs (18,354/22,281), there were 3,927 (17.6%) singleton, which might because of their high uniqueness and species-specific property. To leverage these singletons, we provide users with a sensitive mode in VirBot. In this mode, besides using pHMMs, we conduct pairwise alignment between the query and these singletons by DIAMOND. Once the query protein hits the pHMMs or the DIAMOND database, we will predict it to be RNA viral proteins. To build the DIAMOND database, we used these 3,927 singletons as the positive samples. We also added negative samples to improve the specificity. The negative samples comprise two parts. The first is all DNA viral, prokaryotic, and eukaryotic proteins downloaded from Swiss_Prot. The second is additional bacteria proteins from NCBI RefSeq that share similarities with RNA viral proteins. We searched the bacterial proteins against our pHMMs by hmmsearch (-T 20) first and against the RNA viral singletons by DIAMOND (default) next. All the matched proteins are included in the database. Finally, the DIAMOND database contains 814,991 non-RNA-viral proteins: 559,006 are from Swiss_Prot, and 255,985 are additional bacterial proteins from RefSeq.

2.7 Metrics

We benchmarked various tools by comparing their recall, precision, and F1 score. Recall, measuring the sensitivity of the detection tools, is the ratio of the number of detected RNA viral sequences to the total number of RNA viral sequences. Precision is the ratio of the number of detected RNA viral sequences to the total number of detected sequences, describing the specificity of the tool. F1 score is the harmonic mean of recall and precision.

$$recall = TP/(TP + FN) \tag{2}$$

$$precision = TP/(TP + FP) \tag{3}$$

$$F1 = 2 * recall * precision/(recall + precision) \tag{4}$$

In the above equations, TP denotes true positive, the number of detected RNA viral sequences; FN denotes false negative, the number of missed RNA viral sequences; FP denotes false positive, the number of sequences falsely recognized as RNA viruses.

We defined *accuracy* to evaluate the performance of the taxonomic module. Specifically, we predicted the taxa for the detected contigs, and calculated the percentage of the consistent labels between the prediction and the ground truth as shown in Equation (5). A predicted label is consistent with the known label if they are identical or the predicted label is an ancestor of the known label in the phylogenetic tree.

$$accuracy = \#\ of\ consistent\ prediction/\#\ contigs \tag{5}$$

3 Result

Tool	Command
Kaiju	-t nodes.dmp -f DB.fmi -i FASTA -o RESULT
DIAMOND	diamond blastx –outfmt 6 –max-target-seqs 1 –evalue 1e-5<br>–db DB –query FASTA –out RESULT
MMseqs2	mmseqs easy-search FASTA DB RESULT tmp –greedy-best-hits true
Kraken2	kraken2 –db DB –classified-out RESULT.FASTA –report RESULT FASTA
Virsorter2	virsorter run –provirus-off -w tmp -i FASTA<br>–include-groups "dsDNAphage,NCLDV,RNA,ssDNA,lavidaviridae"
VirFinder	default; score cutoff = 0.9
viralVerify	viralverify -f FASTA -o RESULT –hmm Pfam

Table S2: Commands for benchmarked tools. “DB” refers to the same reference protein database as VirBot’s including all the collected proteins. VirSorter2, VirFinder, and viralVerify use the default model.

To rigorously evaluate the performance of VirBot, we did experiments on both simulated and real sequencing data. We first evaluated the specificity of VirBot on publicly available simulated datasets that are composed of a large

	Raw reads			Assembled contigs				
SRA ID	Read length	# reads	Expected RNA virus abundance[1]	Assembly tool	# contigs (RNA virus)[2] >500bp	# contigs (RNA virus) >1000bp	# contigs (RNA virus) >3000bp	N50
ERR1992810	mixed[3]	13.0M	0.0143	MEGAHIT	161,064 (20)	29,570 (13)	926 (0)	720
ERR1992810	mixed	13.0M	0.0143	SPADES[4]	214,540 (38)	46,636 (23)	1,288 (6)	557
ERR2185279	2*250	12.5M	<0.01%	MEGAHIT	967,818 (18)	264,224 (5)	21,732 (0)	831
ERR2185279	2*250	12.5M	<0.01%	SPADES	887,047 (18)	324,582 (6)	30,035 (0)	1,244

[1] The expected RNA virus abundance is retrieved from NCBI SRA taxonomy analysis.
[2] The number of RNA virus contigs are shown in the parentheses.
[3] ERR1992810 is a mixed dataset with 7.7% single-end reads (1*100bp) and 92.3% paired-end reads (2*100bp).
[4] SPADES runs in –rnaviral mode.

Table S3: Statistics about raw reads and contigs for ERR2185279 and ERR1992810.

number of sequences from non-RNA-viral species. Then, we evaluated the ability of VirBot to detect new RNA viruses in three datasets that contain many novel RNA viruses. Finally, we applied VirBot on RNA virus detection from real metagenomic data sequenced from clinical samples of human. We also evaluated the taxonomic module of VirBot in TOP mode for the simulated datasets and clinical samples, where the true taxonomic labels are known. We benchmarked the performance of VirBot against some popular tools, including DIAMOND [9], MMseqs2 [10], Kraken2 [11], Kaiju [12], VirSorter2 [13], VirFinder [14], and viralVerify [15]. Some of them are generic sequence comparison tools that can be applied to virus detection given our collected RNA viral proteins and we regard the best hit as the prediction result. Some of them are specially designed for virus detection, such as VirSorter2, VirFinder, and viralVerify. We include all virus groups for VirSorter2 and set score cutoff as 0.9 for VirFinder. For a fair comparison, we use the same protein database derived from RNA viruses for those sequence comparison tools and use the default database for those well-complied tools, which already include comprehensive indexes. The commands and parameters of running all the tools are summarized in the Table S2.

3.1 Performance on simulated data

We first evaluated VirBot using a simulated dataset that has a heterogeneous composition of RNA viruses and other species. We downloaded two simulated marine metagenomic samples (NCBI SRA ID: ERR1992810, ERR2185279) [16], which have been used as benchmark datasets for evaluating metagenomic analysis tools. They are semi-synthetic datasets consisting of reads from 82 eukaryotes, 365 prokaryotes, and DNA/RNA viruses. To mimic real marine samples, the data issuers generated the sequencing error profile from real data, integrated the simulated shuffled reads to be unknown reads, and added a low abundance of RNA viral sequences. Because contig assembly can directly affect virus detection performance, we also considered two popular metagenomic assembly programs (MEGAHIT [17] and SPADES). We generated the contigs' labels according to the analysis of NCBI by aligning all the contigs to the identified RNA viruses' genome using megablast. More details about the data are shown in Table S3.

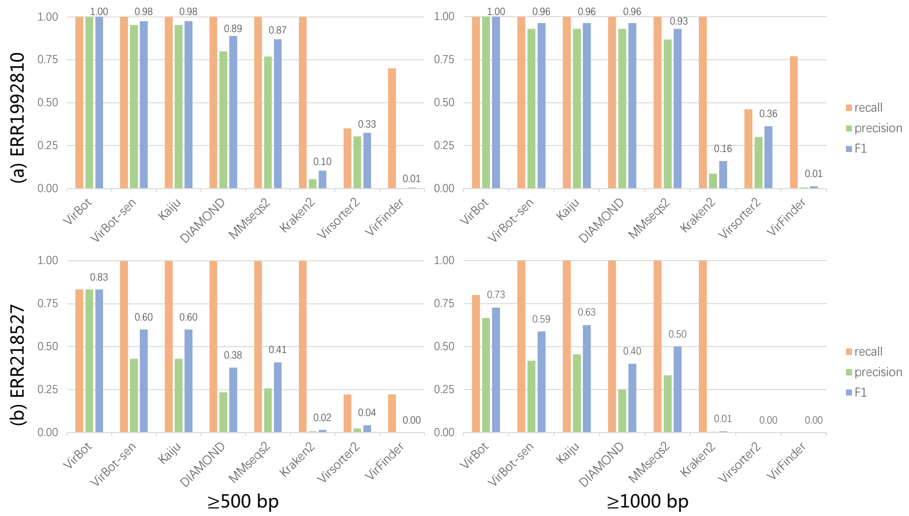

Figure S2: Detection performance on simulated sample ERR1992810 and ERR2185279 (MEGAHIT). The graphs from left to right are for 500bp and 1000bp groups. In ERR2185279, the F1 scores of VirSorter2 and VirFinder are nearly zero because of the low precision.

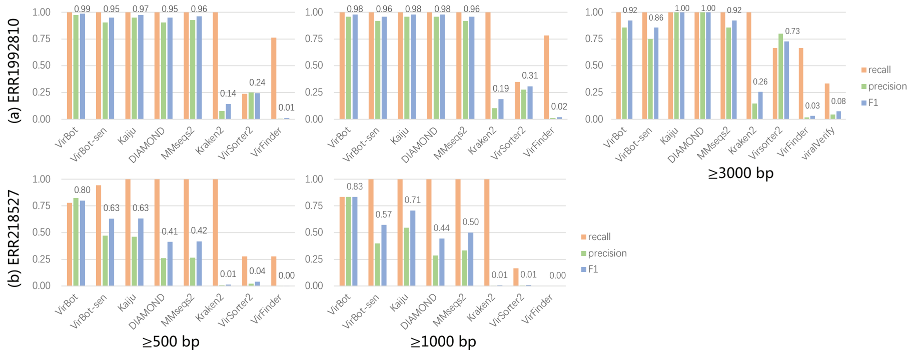

Figure S3: Detection performance on simulated sample ERR1992810 and ERR2185279 (SPADES). The graphs from left to right are for 500bp, 1000bp, and 3000bp groups, respectively. ViralVerify was only evaluated on 3000bp group because it only accepts sequences longer than 3000bp.

We first tested all tools on the contigs assembled by MEGAHIT and recorded their recall, precision, and F1 in Fig. S2. VirBot showed better F1 scores in all groups in these samples. In ERR1992810, VirBot got an F1 score of 100%, outperforming other tools. In ERR2185279, VirBot still showed the highest F1 score of 83.3% and 72.7% in 500bp and 1000bp groups, respectively. Kaiju, the next best tool, achieved an F1 score of 60.0% and 62.5%, respectively. In the sensitive mode, we took advantage of the singletons by integrating a DIAMOND

module, and the recall rate rose to 100% in all samples. In ERR2185279, the 21 false positive contigs were introduced by the diamond module and 3 by HMMER module. And 13 out of 24 contigs were aligned to proto-oncogene or oncogene, which revealed a classification challenge for virus identification.

To examine VirBot's performance under different assemblers, we conducted an extra benchmark experiment by assembling the reads with SPADES's rnaviral mode, which was designed for RNA-seq data and is expected to return more and longer RNA viral contigs. We included viralVerify for the longer RNA viral contigs, because viralVerify can only accept inputs above 3000bp. In ERR1992810, VirBot got the best F1 scores of 98.7% and 97.9% in 500bp and 1000bp group, and ranked second in the 3000bp group (92.3%). The sensitive mode of VirBot returned a few more false positive sequences, resulting in F1 scores of 95.0%, 95.8%, and 85.7%, respectively. In ERR2185279, VirBot showed the highest F1 score of 80.0% and 83.3% in 500bp and 1000bp groups, respectively. For more precise detection, we recommend using the default mode. The result is shown in Fig. S3.

We also tested VirBot on predicting the taxon of each detected contig in its default TOP mode. For those correctly detected RNA virus sequences in ERR1992810 and ERR2185279, VirBot achieved an accuracy of 100% for the taxonomic label assignment.

3.2 Identifying new RNA viruses

To assess the ability of VirBot in identifying new RNA viruses, we used three datasets that contain newly sequenced novel RNA viruses. The first is an RNA phage dataset compiled by Callanan et al. in 2020 [18]. Although the number of sequenced DNA phages has been expanded, we know little about the abundance and diversity of RNA phages. Before 2020, only 12 complete RNA phage genomes were recorded in NCBI RefSeq. To reveal the potential variety of RNA phages, the researchers collected 15,611 non-redundant novel single-strand RNA phage sequences from 82 metatranscriptome samples. This dataset can be downloaded from the supplementary file supplied by the authors. VirSorter2 also used this dataset in their test but did not work well for short sequences ($\leq$3kbp), which is often the case for metagenome-assembled RNA viruses.

The second dataset is an assembled virus-concentrated RNA-sequencing sample collected from Yangshan Deep-Water Harbour, Shanghai, China [19]. The project (NCBI SRA: PRJNA605028) includes both DNA and RNA library. The RNA library was further processed to be an RNA virome sequencing sample. First, the researchers implemented the virus concentration and confirmed the absence of cellular contamination. Then, to filter out the DNA viral particles, they collected the unique 30-mer from the DNA library and excluded all RNA sequences sharing an exact match with these 30-mers. In this way, all remaining particles were RNA viral. Finally, they did the assembly by SPADES –meta [20]. We downloaded the RNA-virus-concentrated assembled dataset from NCBI FTP `https://ftp.ncbi.nih.gov/pub/wolf/_suppl/yangshan/`.

The third is a set of nonredundant sequences containing viral RdRp region

generated by Ahmed et al. in 2022 [21]. To systematically enlarge the marine RNA virus diversity, the researcher collected 771 metatranscriptomes from global oceans. Using an iterative search-and-update method, they identified 5,504 nonredundant RNA virus sequences that encode RdRp gene and are longer than 1,000bp. The phylogenetic analysis showed that these sequences are from all 5 phyla of the RNA virus recoded in ICTV and 3 putative novel phyla. The complex composition enables users to assess their analysis tool comprehensively.

# contig		VirBot	pfam-A[1]
8,849	# detected	7,420	6,627
	recall	83.85%	74.89%

# contig		VirBot	pfam-A
114,139	# detected	43,539	28,970
	recall	38.15%	25.38%

[1] We used the –cut_nc parameter for Pfam, which represents the score of the highest-scoring known false positive and is given by Pfam.

Table S4: Sensitivity of Pfam 35.0 in RNA phage dataset (upper) and marine water RNA virome dataset (lower) (≥500bp).

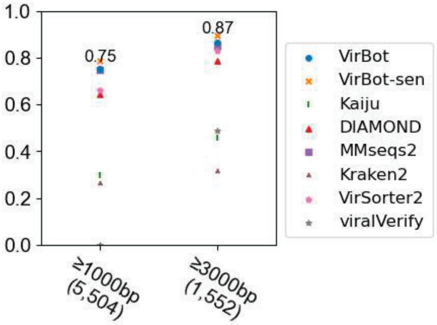

Figure S4: Recall on global oceanic RdRp sequences. The recall rate of VirBot is shown as number.

The result is presented in the Fig. 1(d) and Fig. S4. In different RNA viral datasets (1000bp), VirBot achieved the highest recall rates of 96%, 57%, and 75%, respectively. And in sensitive mode of VirBot, the recall rate slightly rose, which might imply the rareness of the unclustered RNA viral proteins. In the viral RdRp dataset, MMseqs2 got slightly lower recall rate than VirBot, but it detected fewer novel RNA viruses in other two dataset (Fig. 1(d)). The sensitivity of VirBot doesn't just stem from using pHMMs. To validate this, we applied Pfam to the novel RNA virus and the result is shown in Table S4, which clearly shows that just using pHMMs from Pfam does not lead to high sensitivity for RNA virus detection.

For further analysis, we drew the Venn diagrams for the sequences (≥500bp) detected by VirBot two alignment-based methods, DIAMOND and MMseqs2, one alignment-free method, VirFinder, and one hybrid method, VirSorter2. The result is shown in Fig. S5. In RNA phage dataset, these five tools detected 7,865 sequences from 8,849 RNA virus sequences, and VirBot detected 94.3% of the 7,865 sequences. In the marine water RNA virome sample, there were 65,527 detected sequences from 114,139 RNA virus sequences, and VirBot detected 66.4% of the 65,527 sequences. VirBot contributed to the most identified sequences. We then analysed the output agreement between VirBot and other tools. For sequences detected by VirBot, there were 79.6% and 78.1% that could be recognized by three or more tools. The large detection overlaps between VirBot and other tools demonstrate the reliability of VirBot's result. Besides, there were 9.7% and 10.1% of detected sequences uniquely identified by VirBot. On the other hand, although VirFinder exclusively identified 28.8% of detected sequences in

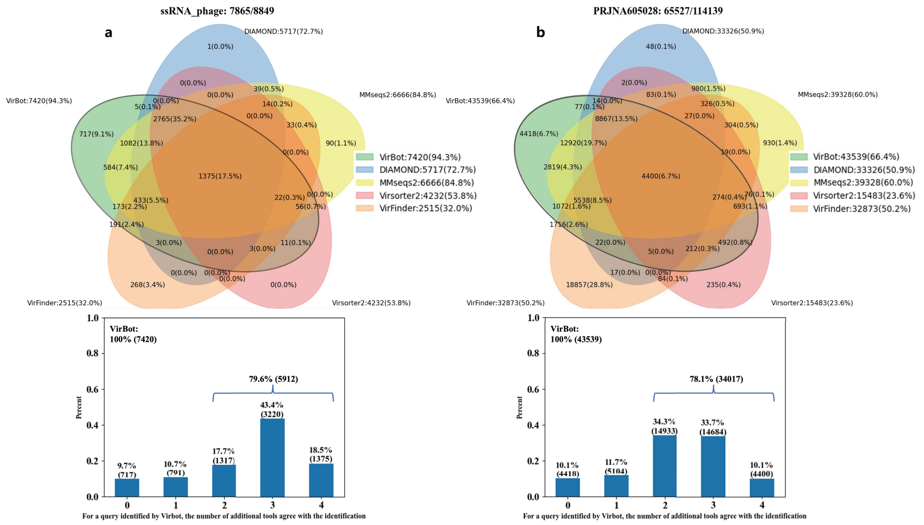

Figure S5: (a) for RNA phage dataset; (b) for marine water RNA virome dataset. The upper graph is Venn diagram of identified sequences in RNA phage dataset (≥500bp) of VirBot, Kaiju, DIAMOND, MMseqs2, Virsorter2, and VirFinder. The lower graph shows the agreement of detection result between VirBot and other tools. For every sequences identified by VirBot, we counted the number of tools that agree with the identification. Each tool that agrees with identification of VirBot will give one point to the identified sequences, and we drew the histogram of the scores for sequences identified by VirBot. For example, in (a), there are 9.7% sequences that identified by VirBot are not identified by other tools, and there are 10.7% sequences that identified by VirBot are identified by another one tool.

the marine water RNA virome sample, its recall rate is limited (52.3% in 3kbp group). This could be attributed to that VirFinder is not optimized for RNA virus. With all the tools, there were 11.1% and 42.6% undetected sequences in two samples, respectively, implying the challenge of new RNA virus detection.

3.3 Performance on real metagenomic data

To evaluate how VirBot performs on identifying viral pathogens from real metagenomic data, we also tested VirBot on human clinical samples. Twenty-one bronchoalveolar lavage fluid sequencing samples published by Shi et al. [22] were exploited. These samples were collected from patients with pneumonia or acute respiratory infections and were confirmed to carry 13 different RNA virus pathogens. To better analyze the bronchoalveolar microbiota, the researchers preprocessed the reads by removing 1) duplicated reads, 2) reads that can be mapped to the human reference genome, and 3) reads that can be mapped to ribosomal

RNA (rRNA). The remaining samples comprised microbial sequences. Then the authors identified the pathogens based on the confirmed diseases and their in-house pipeline.

The abundance of the pathogen can be relatively low and thus we focus on testing whether VirBot can identify these RNA viruses. We tested VirBot's sensitivity for pathogens and specificity in these human samples. To generate the labels, we first aligned all contigs against the pathogens' genomes by megablast to retrieve the pathogens' contigs. Besides the pathogens, other potential RNA viruses were labeled by aligning the identified sequences against nt/nr by megablast/blastx. If a contig does not match to any nucleotide sequences but matches to RNA viral proteins, we treat it as novel RNA virus sequences. The chimeric contigs sharing the identical region with both bacteria and virus were removed from the evaluation. We set the minimum contigs' length as 500bp.

VirBot successfully retrieved all the reported RNA viral pathogens. Besides the confirmed RNA viral pathogens, VirBot also detected some unreported known RNA viruses and novel RNA virus sequences. VirBot achieved an average recall of 99.3% and precision of 99.2% among different groups. For those detected RNA viral pathogens, we evaluated the accuracy of the taxonomic assignment of VirBot. 25.2% of these contigs were assigned at *species* rank, 21.4% at *genus*, and 52.4% at *family.* It achieved an average accuracy of 94.9%, showing its potential applicability in clinical diagnosis. The accuracy of taxonomic assignment for each sample is shown in Table S5. In the influenza B virus (IVB) samples, the accuracy dropped to 42.9% and 50%, because VirBot misclassified some segments of IVB as IVA, which is in the same family as IVB. VirBot also detected novel RNA viral sequences that shared little similarity with known nucleotide sequences and got 140 in total. The result is shown in Table S5.

4 Discussion

RNA viruses are highly diverse, making RNA virus detection in metagenomic data difficult. Previously, conserved protein families have been successfully employed for novel bacteria detection. But the lack of marker genes inhibits its further application in RNA virus detection. VirSorter2 and viralVerify leverage the RdRp and Pfam. But the limited number of RNA viral pHHMs jeopardizes their sensitivity of RNA virus detection. In this work, we constructed a comprehensive protein family database for known RNA viruses and enlarged the number of applicable RNA viral pHMMs by 1384. We validated their distinguishing power by examining their hit score distributions for RNA viruses and a large number of non-RNA viruses. Considering that some RNA viral proteins share homologs with proteins from DNA virus and cellular species, we set adaptive cutoff for each protein family instead of using a fixed cutoff. Using the adaptive bit score cutoff, we developed VirBot based on the RNA viral pHMMs. Compared with using a fixed cutoff (such as E-value), the adaptive bit score cutoff improved the specificity from 8.8% to 97.4% in the simulated data, which is shown in Table

Sample	Pathogen	# contig	# detected contigs	Recall[1]	Precision	# novel RNA viral contigs	Acc of taxa[2]
S0408QHG	ENV-D	567	16	85.7%	100.0%	1	100.0%
S0045ZHW	ENV-D	265	5	100.0%	100.0%	0	100.0%
S0679ZYY	HRV	314	7	100.0%	100.0%	2	100.0%
S0939LX	HRV,HPIV3	2335	12	100.0%	100.0%	6	100.0%
S0988LHQ	HRV	6674	27	100.0%	100.0%	19	100.0%
S0876LGD	HRV	3829	39	100.0%	100.0%	13	100.0%
S0560WT	IVA	202	18	100.0%	100.0%	2	100.0%
S0838XG	IVA	4366	31	100.0%	100.0%	19	100.0%
S0846ZYL	IVB	4591	46	100.0%	100.0%	25	42.9%
S0956HCE	IVB	18747	28	100.0%	96.4%	16	50.0%
S0594ZCJ	HPIV1	2473	3	100.0%	100.0%	0	100.0%
S0723HZX	HPIV3	278	16	100.0%	100.0%	6	100.0%
S0725PCX	HPIV4	948	8	100.0%	87.5%	0	100.0%
S0579HDY	HPMV,HRV	3813	4	100.0%	100.0%	0	100.0%
S0574ZZX	RSV	644	10	100.0%	100.0%	3	100.0%
S0970XLQ	229E	12562	13	100.0%	100.0%	11	100.0%
S0677SP	229E	838	7	100.0%	100.0%	1	100.0%
S0959FBW	229E,RSV	6357	19	100.0%	100.0%	4	100.0%
S0069ZH	NL63	769	24	100.0%	100.0%	0	100.0%
S0979LY	OC43	3362	17	100.0%	100.0%	12	100.0%
S0561YYM	HKU1	2283	15	100.0%	100.0%	0	100.0%

[1] The recall is calculated for pathogen contigs.
[2] The accuracy of taxonomic assignment.

Table S5: Detection result of VirBot on clinical samples.

S6. Moreover, we embedded a taxonomic module in VirBot, which achieved an average accuracy of 94.9% in human RNA viral pathogens detection.

We investigated the 19 pHMMs that have bit score distribution overlap between RNA virus and other species. These pHMMs are from protein domains/families shared by RNA virus and cellular organisms. The prokaryotic (reverse) transcriptase, DEAD/DEAH box helicase, the oncogene/proto-oncogene, and the GTPase are four kinds of proteins that dominate the misidentified sequences. The DEAD/DEAH box helicase is involved in various aspects of RNA metabolism [23]. The oncogene/proto-oncogene has been demonstrated to be evolutionary related to viruses [24]. And the GTPase, proteins hydrolyzing GTP, is associated with the basic biological signal transduction [25]. Besides, VirBot identified some endogenous retroviral proteins from eukaryotic sequences. These proteins might be the viral heritage during the long-time evolution. To improve the specificity, we regarded them as non-viral sequences. How to identify and retrieve the retrovirus sequences from host deserves further studies.

The enlarged RNA viral pHMM collection and the application of adaptive cutoffs improve the accuracy of RNA virus identification. In particular, VirBot shows better sensitivity and specificity than both alignment-based and learning-based tools. However, VirBot still has several limitations. First, the default gene prediction of VirBot is conducted by Prodigal. We did not compare its performance with other gene prediction tools designed for viruses. The splicing

Sample (assembler): # contigs (# RNA viruses)	ERR1992810 (MEGAHIT): 161,064 (20)		ERR1992810 (SPADES): 214,540 (38)		ERR2185279 (MEGAHIT): 967,818 (18)		ERR2185279 (SPADES): 887,047 (18)	
	Adaptive cutoff	Fixed cutoff	Adaptive cutoff	Fixed cutoff	Adaptive cutoff	Fixed cutoff	Adaptive cutoff	Fixed cutoff
# detected contigs	20	363	39	431	18	2578	17	2640
# detected RNA viruses	20	20	38	38	15	15	14	14
recall	100.0%	100.0%	100.0%	100.0%	83.3%	83.3%	77.8%	77.8%
precision	100.0%	5.5%	**97.4%**	**8.8%**	83.3%	0.6%	82.4%	0.5%
F1	100.0%	10.4%	98.7%	16.2%	83.3%	1.1%	80.0%	1.1%

Table S6: The detection result of VirBot using either adaptive cutoff or fixed cutoff (E-value of $1e^{-10}$) on the simulated data (≥500bp).

events or different predictions can affect the alignment scores and thus new RNA virus detection. Second, although our current pHMMs are derived from all sequenced RNA viruses, they cannot match novel proteins of new RNA viruses. If a contig from a new RNA virus contains only new proteins, it will be missed by VirBot and also other tools. With the increased RNA virus database and their associated annotations, this problem will be mitigated to some extent. It will be interesting to conduct a networking analysis to examine the conservation of all these protein families in our future work.

References

[1] Doug Hyatt, Gwo-Liang Chen, Philip F LoCascio, Miriam L Land, Frank W Larimer, and Loren J Hauser. Prodigal: prokaryotic gene recognition and translation initiation site identification. BMC bioinformatics, 11(1):1–11, 2010.

[2] Limin Fu, Beifang Niu, Zhengwei Zhu, Sitao Wu, and Weizhong Li. CD-HIT: accelerated for clustering the next-generation sequencing data. Bioinformatics, 28(23):3150–3152, 2012.

[3] shenwei356 pirovc. genome_updater. https://github.com/pirovc/genome_updater, 2019.

[4] Jaina Mistry, Sara Chuguransky, Lowri Williams, Matloob Qureshi, Gustavo A Salazar, Erik LL Sonnhammer, Silvio CE Tosatto, Lisanna Paladin, Shriya Raj, Lorna J Richardson, et al. Pfam: The protein families database in 2021. Nucleic Acids Research, 49(D1):D412–D419, 2021.

[5] Wolfgang Gerlach and Jens Stoye. Taxonomic classification of metagenomic shotgun sequences with CARMA3. Nucleic Acids Research, 39(14):e91–e91, 2011.

[6] Anton J Enright, Stijn Van Dongen, and Christos A Ouzounis. An efficient algorithm for large-scale detection of protein families. Nucleic Acids Research, 30(7):1575–1584, 2002.

[7] Kazutaka Katoh, Kazuharu Misawa, Kei-ichi Kuma, and Takashi Miyata. MAFFT: a novel method for rapid multiple sequence alignment based on fast fourier transform. Nucleic Acids Research, 30(14):3059–3066, 2002.

[8] Sean R. Eddy. Profile hidden Markov models. Bioinformatics, 14(9):755–763, 1998.

[9] Benjamin Buchfink, Chao Xie, and Daniel H Huson. Fast and sensitive protein alignment using DIAMOND. Nature Methods, 12(1):59–60, 2015.

[10] Martin Steinegger and Johannes Söding. MMseqs2 enables sensitive protein sequence searching for the analysis of massive data sets. Nature Biotechnology, 35(11):1026–1028, 2017.

[11] Derrick E Wood, Jennifer Lu, and Ben Langmead. Improved metagenomic analysis with Kraken 2. Genome Biology, 20(1):1–13, 2019.

[12] Peter Menzel, Kim Lee Ng, and Anders Krogh. Fast and sensitive taxonomic classification for metagenomics with Kaiju. Nature Communications, 7(1):1–9, 2016.

[13] Jiarong Guo, Ben Bolduc, Ahmed A Zayed, Arvind Varsani, Guillermo Dominguez-Huerta, Tom O Delmont, Akbar Adjie Pratama, M Consuelo Gazitúa, Dean Vik, Matthew B Sullivan, et al. VirSorter2: a multi-classifier, expert-guided approach to detect diverse DNA and RNA viruses. Microbiome, 9(1):1–13, 2021.

[14] Jie Ren, Nathan A Ahlgren, Yang Young Lu, Jed A Fuhrman, and Fengzhu Sun. VirFinder: a novel k-mer based tool for identifying viral sequences from assembled metagenomic data. Microbiome, 5(1):1–20, 2017.

[15] Dmitry Antipov, Mikhail Raiko, Alla Lapidus, and Pavel A Pevzner. Metaviral SPAdes: assembly of viruses from metagenomic data. Bioinformatics, 36(14):4126–4129, 2020.

[16] Alex Mitchell, Maxim Scheremetjew, Gianluca De Moro, Bruno Fosso, Monica Santamaria, Graziano Pesole, Shriya Raj, Lars Ailo Bongo, Nils Peder Willassen, and Robert D. Finn. ELIXIR-EXCELERATE D6.3: Report describing a set of tools, pipelines and search engine for interrogation of marine metagenomic data., 2019.

[17] Dinghua Li, Chi-Man Liu, Ruibang Luo, Kunihiko Sadakane, and Tak-Wah Lam. MEGAHIT: an ultra-fast single-node solution for large and complex metagenomics assembly via succinct de Bruijn graph. Bioinformatics, 31 (10):1674–1676, 2015.

[18] Julie Callanan, Stephen R Stockdale, Andrey Shkoporov, Lorraine A Draper, R Paul Ross, and Colin Hill. Expansion of known ssRNA phage genomes: from tens to over a thousand. Science Advances, 6(6):eaay5981, 2020.

[19] Yuri I Wolf, Sukrit Silas, Yongjie Wang, Shuang Wu, Michael Bocek, Darius Kazlauskas, Mart Krupovic, Andrew Fire, Valerian V Dolja, and Eugene V Koonin. Doubling of the known set of RNA viruses by metagenomic analysis of an aquatic virome. Nature Microbiology, 5(10):1262–1270, 2020.

[20] Sergey Nurk, Dmitry Meleshko, Anton Korobeynikov, and Pavel A Pevzner. metaSPAdes: a new versatile metagenomic assembler. Genome Research, 27(5):824–834, 2017.

[21] Ahmed A Zayed, James M Wainaina, Guillermo Dominguez-Huerta, Eric Pelletier, Jiarong Guo, Mohamed Mohssen, Funing Tian, Akbar Adjie Pratama, Benjamin Bolduc, Olivier Zablocki, et al. Cryptic and abundant marine viruses at the evolutionary origins of Earth's RNA virome. Science, 376(6589):156–162, 2022.

[22] Mang Shi, Su Zhao, Bin Yu, Wei-Chen Wu, Yi Hu, Jun-Hua Tian, Wen Yin, Fang Ni, Hong-Ling Hu, Shuang Geng, et al. Total infectome characterization of respiratory infections in pre-COVID-19 Wuhan, China. PLoS pathogens, 18(2):e1010259, 2022.

[23] Ourania Andrisani, Qian Liu, Patricia Kehn, Wolfgang W Leitner, Kyung Moon, Nancy Vazquez-Maldonado, Ian Fingerman, and Michael Gale. Biological functions of DEAD/DEAH-box RNA helicases in health and disease. Nature Immunology, 23(3):354–357, 2022.

[24] Zhi-Ming Zheng. Viral oncogenes, noncoding RNAs, and RNA splicing in human tumor viruses. International Journal of Biological Sciences, 6(7): 730, 2010.

[25] Emi Mizuno-Yamasaki, Felix Rivera-Molina, and Peter Novick. GTPase networks in membrane traffic. Annual Review of Biochemistry, 81:637, 2012.
